# Understanding Sex-biases in Kinetoplastid Infections: Leishmaniasis and Trypanosomiasis

**DOI:** 10.1017/erm.2024.41

**Published:** 2025-01-09

**Authors:** Olivia Battistoni, Ryan H. Huston, Chaitenya Verma, Thalia Pacheco-Fernandez, Sara Abul-Khoudoud, Alison Campbell, Abhay R. Satoskar

**Affiliations:** 1 Department of Pathology, Wexner Medical Center, The Ohio State University, Columbus, OH, USA; 2 Department of Microbiology, Wexner Medical Center, The Ohio State University, Columbus, OH, USA; 3 Department of Biotechnology, Sharda School of Engineering & Technology, Sharda University, Greater Noida, UP, India; 4 Division of Emerging and Transfusion Transmitted Disease, Center for Biologics Evaluation and Research, U.S. Food and Drug Administration, Silver Spring, MD, USA

**Keywords:** African Trypanosomiasis, behavior, Chagas disease, genetics, hormones, immune response, *Leishmania*, Leishmaniasis, neglected tropical disease, sex-bias infection

## Abstract

**Background:**

Leishmaniasis, Chagas disease (CD), and Human African Trypanosomiasis (HAT) are neglected tropical diseases in humans caused by intracellular parasites from the class *Kinetoplastida*. Leishmaniasis is one infectious disease that exhibits sex-bias not explained solely by behavioral or cultural differences. However, HAT and CD have less well documented and understood sex-related differences, either due to a lack of differences or insufficient research and reporting.

**Methods:**

This paper reviews the rate of disease and disease severity among male and females infected with CD, HAT, and leishmaniasis. We further review the specific immune response to each pathogen and potential sex-based mechanisms which could impact immune responses and disease outcomes.

**Results:**

These mechanisms include sex hormone modulation of the immune response, sex-related genetic differences, and socio-cultural factors impacting risky behaviors in men and women. The mechanistic differences in immune response among sexes and pathogens provide important insights and identification of areas for further research.

**Conclusions:**

This information can aid in future development of inclusive, targeted, safe, and effective treatments and control measures for these neglected diseases and other infectious diseases.

## Introduction

Kinetoplast parasites are a group of flagellated protozoans defined by possession of the kinetoplast organelle. As a group, these organisms infect plants and animals (Refs 1, 2, 3, 4, 5). However, three kinetoplastid species – *Trypanosoma cruzi* (*T.c.*), *Trypanosoma brucei* (*T.b.*) and *Leishmania spp.* – cause human diseases recognized by the World Health Organization (WHO) as Neglected Tropical Diseases (NTDs) (Ref. 6). These include Chagas disease (CD), human African trypanosomiasis (HAT) and leishmaniasis, respectively. Each of the three human kinetoplastid diseases has a life cycle involving a blood-feeding arthropod vector as well as a human host and express variable sex-bias in their incidence, morbidity and mortality rates. In this review, we will share evidence of such sex-biases in these diseases as well as explore potential mechanisms and areas requiring further research especially surrounding the immune response, sex-hormone impacts and genetic concerns.

## Etiology and epidemiology

**Figure 1.**
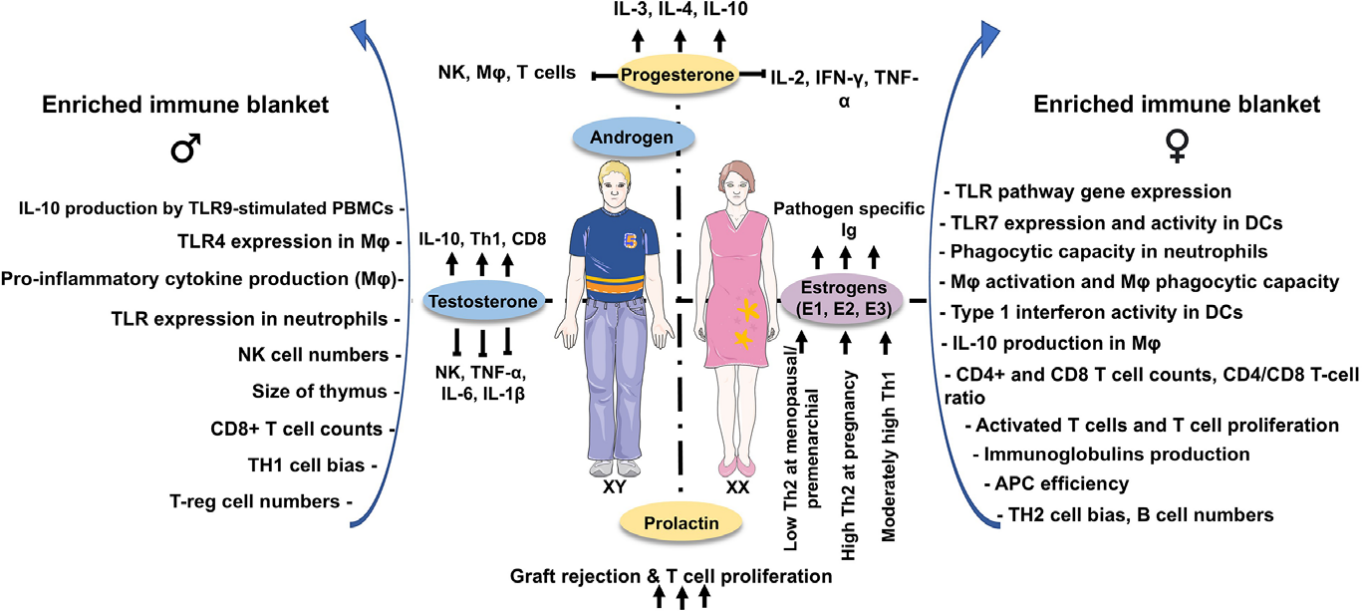
Hormone differences and immune cell effects between XY males and XX females. While hormone levels vary from person to person, the centre of this figure depicts from left (male) to right (female) typical relative levels of sex hormones between males and females in sexually mature individuals. The left sided text describes typical immune cell effects of increased male sex hormones, and the right sided text describes typical immune cell effects of increased female sex hormone levels. Some immune effects of sex hormones have more complex non-linear relationships between hormone concentration and immune response, which are not fully depicted in this figure.

**Figure 2.**
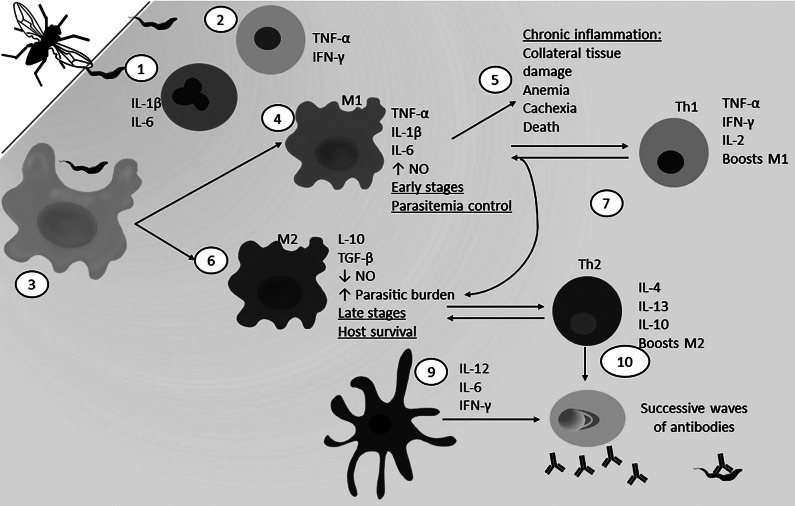
Immune response against *Trypanosoma brucei. T. brucei* is transmitted by the bite of many species of tsetse flies belonging to the genus *Glossina.* Thus, initial immune response happens in the skin, where first responder cells such as (1) neutrophils (purple) and (2) NK cells (light blue) elicit inflammatory cytokines which will later favour macrophage activation towards M1 (Refs 97, 98). At early stages of infection, (3) macrophages (beige) play an important role phagocyting *T. brucei* (Ref. 99). (4) Macrophage polarization towards M1 (orange) controls parasitaemia in the early stages of the infection, mainly through the production of TNF-α and NO. (5) Nevertheless chronic inflammation becomes harmful for the host and can cause collateral tissue damage, anaemia, cachexia and even death (Refs 100, 101). (6) Therefore, macrophage polarization shift from M1 to M2 is necessary for host survival (Refs 100, 101). Although an exacerbated M2 prevalence leads to increased parasitic burden. (7) M1 and M2 cells sustain and are favoured by the Th1 and Th2 cells (green), respectively. (8) Moreover, IL-4 from Th2 cells, (9) together with IL-12, IL-6 and IFN-γ produced by the dendritic cells (blue), induce the antibody production by plasma B cells (pink) (Refs 97, 98). 10) Finally, due to parasite antigenic variability and its capacity to survive extracellularly, multiple successive waves of anti-*T.b.* antibodies are generated by plasma B cells (pink) (Ref. 101). Therefore, those antibodies can direct phagocytosis, necessary for long-term parasitaemia control (Ref. 101).

**Figure 3.**
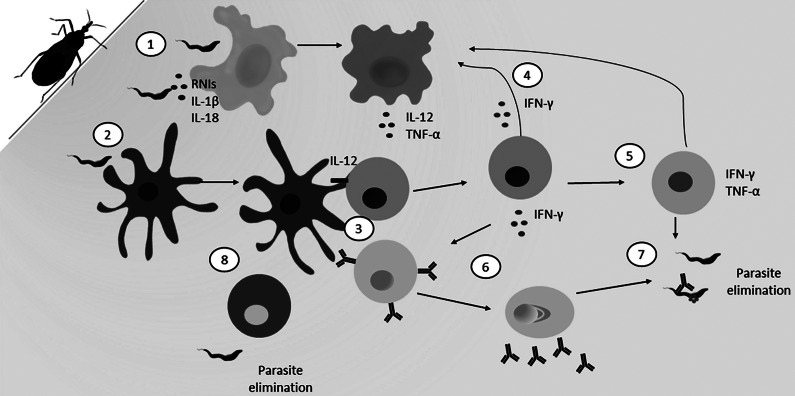
Immune response against *Trypanosoma cruzi. Trigonoscuta cruzi* is usually transmitted by the bite of a triatomine vector (also known as kissing bug). The infection is then divided in acute and chronic stages (Ref. 112). During the acute phase (1) macrophages (beige) are recruited to the site of infection and are capable of phagocyting the parasite, releasing reactive nitrogen intermediates (RNI) and promoting inflammasome formation and IL-1β and IL-18 cytokines that favours the parasite elimination (Ref. 112). (2) Similarly, dendritic cells (blue) also phagocytize the parasite and migrate to the draining lymph node for antigen presentation to the T and B cells. (3) T cell (green) Th1 phenotype is sustained by cytokine production of activated macrophages and dendritic cells, such as IL-12 and TNF-α (Refs 113, 114). 4) Th1 cells are IFN-γ producers which sustains (4) classical activation of the macrophages, (5) and promotes NK cells’ (light blue) trypanocidal effector activity and macrophage activation (Ref. 112). Moreover, (6) IFN-γ stimulates IgG2a production by plasma B cells (pink) (Refs 112, 114). (7) Antibodies opsonize the parasite and favour one of the three occurring complement cascades (red), resulting in parasite elimination (Ref. 113). (8) During the chronic asymptomatic phase of the disease, cytotoxic CD8+ T cells (yellow) are critical for parasitaemia control via cytokine secretion and infected cells elimination (Ref. 113).

**Figure 4.**
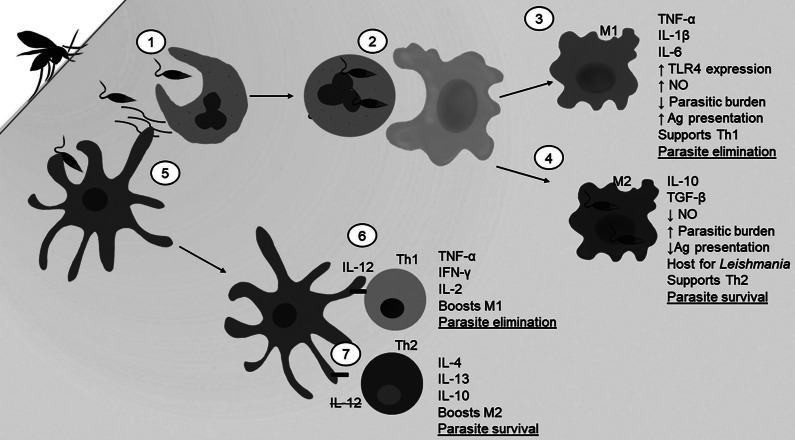
General immune response against leishmaniasis. *Leishmania spp.* can be transmitted by different species of phlebotomine sandflies. Although *Leishmania* infection immune response differs among the different species (Ref. 132), in general (1) neutrophils (purple) are the first immune cells to arrive to the sandfly bite site. There, neutrophils will release granules and NETs which favour parasite elimination (Refs 133, 134). Nevertheless, they can also act like Trojan horses, internalizing parasites and helping them to reach their definitive host, the macrophage (beige) (Refs 135, 136). (2) Macrophages then phagocytize the infected neutrophils and become infected themselves (Ref. 136). The immune polarization of the macrophage will determine the course of the infection (Ref. 137). 3) Inflammatory macrophages (Ref. 138) (orange), characterized by higher TLR4 expression as well as by the production of nitric oxide (NO) and inflammatory cytokines as TNF-α, IL-1β and IL-6 will reduce parasite burden and increase antigen presentation. Additionally, M1 macrophages will support Th1 response later, overall resulting to parasite elimination. On the other hand, (4) anti-inflammatory macrophages (M2) (brown) show a reduction in ROS and produce IL-10 and TGF-β, inhibiting inflammatory response and permitting parasite survival, moreover, such cells become long-term host for *Leishmania* (Ref. 132). (5) At the site of infection, dendritic cells (DCs) (blue) will phagocytize the parasite and take it to the lymph node to be presented to T lymphocytes (green). T cells can differentiate into (6) Th1 through the secretion of IL-12 by the DC which leads to the production of TNF-α, IFN-γ and IL-2 which supports Th1 cells proliferation and boost M1 activation, leading to parasite elimination. (7) In absence of IL-12, T cells may also differentiate into Th2 which supports M2 activation though the cytokines IL-4, IL-13 and IL-10, promoting parasite survival (Ref. 137). Whereas effector Th1 and Th2 cells play a direct role in parasite elimination or persistence, long term immunity is sustained by memory T cells.

Table 1 summarizes the transmission, epidemiology, clinical manifestations, current treatments and prevention strategies for each of the three diseases that will be explored in this paper. This is a general overview and does not cover fine details of each of these categories. The cellular immune responses to each of these disease processes are later detailed in Figures 1, 2, 3, and 4.

**Table 1.** Etiology and Epidemiology of Leishmaniasis, Chagas disease and Human African Trypanosomiasis. Overview of the modes of transmission, areas of endemicity, rates of disease, clinical manifestations and current treatment and prevention strategies

Disease	Transmission	Region of Endemicity	Incidence and Mortality	Clinical Manifestations	Treatment and Prevention
Leishmaniasis	20 species of *Leishmania* are transmitted to humans mainly through bite of a sandfly (Ref. 234).	Tropical and subtropical countries, especially the Mediterranean, Middle East, East Africa, South Asia and Central and South America (Ref. 235).	Global prevalence is estimated at 4–10 million cases depending on year, while there are 700,000–1.2 million new cases per year, and 70,000 deaths per year (Refs 3, 236).	There are three main forms (Refs 237, 238). *Visceral Leishmaniasis* is a systemic infection especially in the spleen, liver and bone marrow and can be fatal (Ref. 239). *Cutaneous Leishmaniasis* yields lesions limited to the skin with various clinical severities (Ref. 239). *Mucocutaneous Leishmaniasis* results in destructive nasopharyngeal lesions, permanent disfiguration, and other complications (Refs 237, 239).	Optimal treatment varies but includes intravenous L-Amphotericin B and oral miltefosine which are FDA approved treatments (Refs 240, 241). No vaccine is commercially available but currently there are candidate human vaccines in early phase clinical trials (Refs 242, 243, 244, 245). Prevention otherwise centres around treatment of cases and vector control (Refs 246, 247).
Chagas Disease	*T.c.* is commonly spread through infected feces of the nocturnal triatomine bug (Refs 248, 249, 250).	Transmission occurs mostly in Latin America (Ref. 251), but due to migration detection also occurs at substantial levels in non-endemic countries (Refs 248, 252).	Prevalence estimated at 6–7 million individuals, around 100,000 new cases per year, and 10,000 deaths annually (Refs 6, 248).	CD impacts the central nervous, gastrointestinal and cardiovascular systems, with the latter leading to mortality (Ref. 253). Symptoms vary by phase. *Acute phase infection*: Fever, oedema, lymphadenopathy, anaemia, hepatosplenomegaly, cardiac abnormalities, central nervous system involvement and death (Refs 47, 250, 251, 252, 253). *Indeterminate phase infections*: asymptomatic (Refs 250, 254). *Chronic phase infections*: Fever, cardiomegaly, apical aneurysms, ECG abnormalities and cardiac involvement and gastrointestinal tract involvement (Refs 47, 250, 251, 252, 253).	Treatments include benznidazole and nifurtimox for acute infections and symptom management for related cardiac disease (Refs 255, 256, 257). There is not yet a commercially available vaccine, although research is ongoing (Refs 258, 259).
Human African Trypanosomiasis	*T.b.* is mainly spread by the bite of an infected tsetse fly (Refs 58, 59).	Distributed throughout sub-Saharan Africa, with highest rates of transmission in rural areas (Ref. 226). The highest incidence occurs in the Democratic Republic of the Congo with 522 cases/year. All other African countries report between 0–100 cases per year (Refs 226, 260).	Due to control efforts, now less than 1000 new cases reported each year (Refs 59, 256). Of the two forms of disease caused by two different subspecies of parasite, West African Trypanosomiasis (92–98% of cases) is much more prevalent compared to east African Trypanosomiasis (2–8% of cases) (Refs 59, 256, 257). No recent reports of mortality rates have been published.	HAT is a progressive and usually fatal if untreated (Refs 257, 258). Presents with skin reaction with or without lymphadenopathy near the bite site. Subsequent systemic disease initiates via blood but manifestation is subspecies dependent. *West African trypanosomiasis*: Stage one begins with irregular fever but can be otherwise asymptomatic or also have hepatosplenomegaly and lymphadenopathy (Refs 261, 262). *East African trypanosomiasis*: Also begins with irregularly fever, but this disease form is more severe. 10% of infections at stage one are fatal - usually attributed to myocardial involvement (Refs 261, 262). *Stage two for both subspecies*: Infection spreads to cerebral spinal fluid (CSF) leading to neurological symptoms such as sleep cycle disruptions, ataxia, confusion, coma and eventually death (Refs 59, 261, 262).	In the absence of a vaccine, treatment strategies for HAT include nifurtimox–eflornithine combination therapy (NECT) and fexinidazole; previous treatment methods such as melarsoprol are no longer in use due to significant toxicities (Refs 260, 261).

**Abbreviations:** *T.c.*: *Trypanosoma cruzi*; CD: Chagas disease; *T.b*: *Trypanosoma brucei*; HAT: Human African Trypanosomiasis; NECT: nifurtimox–eflornithine combination therapy.

## Sex bias: epidemiology and clinical presentation

The precise global incidence and morbidity rates of neglected tropical diseases are difficult to quantify given that inherent constraints in diagnosis and clinical management cause many cases to remain undiagnosed (Ref. 7). For infectious diseases in general however, sex is known to alter the rate of infection, disease course and severity – often presenting with higher rates of morbidity and mortality in males than in females (Ref. 8). Based on current evidence, *Leishmania*, *T.c.* and *T.b.* exhibit differences in the distribution of disease between sexes despite each parasite’s evolutionary relatedness.

Some parasitic infectious diseases exhibit clear male predominance in disease severity, such as *Entamoeba histolytica* (Ref. 9), while in other parasitic infections, evidence of sex bias effects remains inconclusive or is limited to one stage of infection (Ref. 10. Sex-bias in infection and clinical manifestations is related to several factors including genetics, hormones and gender-specific behaviours. A variance in sex-bias has been observed across age in certain infectious diseases, primarily associated with known age-related fluctuations in sex hormone levels, which depicts the interplay of hormones and the immune system in the clinical presentation of sex bias in infectious diseases (Ref. 8). Sex biases with female predominance are present in non-infectious autoimmune conditions, including multiple sclerosis (MS) (Ref. 11), systemic sclerosis (Ref. 12), Hashimoto’s thyroiditis (Ref. 13), myasthenia gravis (MG) (Ref. 14), rheumatoid arthritis (RA) (Ref. 15), systemic lupus erythematous (SLE) (Ref. 16), among others. In addition to having a female bias, many of these autoimmune conditions, such as MS, MG, SLE and RA, exhibit changes in symptoms and severity with variations in the hormonal state of the host such as puberty, pregnancy and menopause (Ref. 17). This further emphasizes the role that sex differences such as hormones play in the immune response.

Sex biases following patterns seen with other infectious diseases have been observed in infection by kinetoplastid parasites. As covered in the following sections, evidence suggests these kinetoplastid diseases demonstrate greater morbidity and mortality among infected males, though inconsistencies exist between reports. We will examine the current data on sex differences in incidence, progression and severity for each disease individually.

### Leishmaniasis

Leishmaniasis has been established as exhibiting sex-bias with higher incidence and worse disease outcomes in males by both survey data and medical records, with the male-to-female ratio of disease increasing with increased age (Refs 18, 19, 20, 21, 22, 23, 24, 25, 26). A local study in Israel, a country with endemic Old-World cutaneous leishmaniasis (CL), found female incidence of leishmaniasis to be about 3/4 of that in males. Furthermore, another study reported a relative risk of 1.27 of developing visceral leishmaniasis (VL) when comparing males to females (Ref. 27). Clinical variations between the sexes in instances of infection have been well-defined but are variable between specific *Leishmania spp.* among other factors (Ref. 10). Previous studies show differences in infectivity dependent on the level of sex hormones in the host (Ref. 10), species and strain of the model (Ref. 28), parasite strain (Ref. 28) and route of inoculation (Ref. 29). Thus, the age, host and parasite are important factors to consider when exploring the differences between sexes associated with leishmaniasis infection.

Cutaneous leishmaniasis in animal models is more severe in male organisms compared to females, with males experiencing larger lesion sizes (Ref. 29), higher parasite burdens (Refs 28, 29) and less inflammatory responses compared to females (Ref. 10). Specifically, these observations were made with *L. major*, *L. mexicana*, *L. panamensis* and *L. guyanenis* rodent infection models (Refs 10, 28, 29). Male mice infected with *L. major* have been shown to have a significantly greater parasite burden in the liver (Ref. 28) and lymph nodes (Ref. 29) compared to females. Nevertheless, no significant differences exist between male and female hamsters for neither *Leishmania*-specific antibody titres nor in the delayed-type hypersensitivity responses (Ref. 29). Differences in disease-promoting cytokines present at the infection site have been noted, with lesions in males having significantly increased interleukin (IL)-10, IL-4 and transforming growth factor (TGF)-β cytokine levels which are known to be associated with larger and more severe lesions (Ref. 29). Similarly, as in the animal models, differences in leishmaniasis severity are being observed in humans by gender also.

In humans, data shows higher rates of CL reported amongst males compared to females, with males having a higher rate of subsequent progression to mucocutaneous leishmaniasis (MCL). Although studies have been limited, evidence has shown male patients are more likely to suffer from CL caused by *L. major, L tropica* and *L guyanensis* (Refs 30, 31, 32, 33). Other single region studies found CL caused by *L. tropica* and *L. major* had no significant sex-bias (Refs 34, 35) partially in contrast to the animal models described above. Moreover, an Afghanistan-based study found that *L. tropica* infection caused increased CL lesions and scarring in females than in males (Ref. 34). Likewise in VL caused by *Labrus donovani* and *L. infantum*, the male sex has been identified as a risk factor for infection (Refs 25, 36, 37, 38, 39, 40, 41), although most studies investigating disease severity in humans have been limited by a single-location study design or by a low number of cases (Ref. 24). Studies have also found experience worse severity of VL infection, although when stratified by age, this trend is present only in sexually mature males over the age of 15 (Refs 23, 27, 42). Younger pre-pubescent females, below age 15, exhibited higher rates of VL-related mortality compared to males under 15 (Ref. 24). Notably, trends surrounding disease severity of VL do not universally correlate with sex. One analysis of suspected cases of VL in India found male-dominant VL disease incidence, but no significant difference in mortality rates between males and females (Ref. 24). This finding may be due to increased healthcare access and thus disease reporting in males, with reporting limited to severe VL cases in females, or due to female cases more commonly being asymptomatic and thus unreported, as was discussed in the study (Ref. 24). These contrasting results indicate a need for further research into the variance of disease severity of VL between males and females using larger patient groups, testing and inclusion of asymptomatic individuals and records of disease progression for a better understanding of sex-bias in symptomatic VL.

### Chagas disease

Some reports suggest Chagas disease may exhibit higher incidence in males compared to females (Refs 43, 44, 45), while others have found the difference to be insignificant (Ref. 46), indicating a need for better diagnosis, documentation and reporting of cases to better understand the presence or lack of sex-bias in disease incidence of CD. CD-related cardiac disease and irregularities are significantly more prevalent in male patients compared to females (Ref. 43). However, information regarding clinical sex differences in *T.c.* infection is complicated by the fact that many cases remain in the intermediate, or asymptomatic, stage and clinical sequelae of CD, such as cardiomyopathies, are not unique to this disease and thus may not be attributed to CD if the underlying infection is not detected (Refs 47, 48). While detecting differences in CD severity in humans is challenging, animal models also support this observation of increased disease severity and complications of CD in males compared to females. Mouse models demonstrate greater susceptibility to infection, as demonstrated by higher parasitaemia levels, and a higher rate of mortality in males compared to females (Refs 49, 50).

Investigations into the role of sex in the progression of CD in humans have largely focused on cardiomyopathy in *Trigonoscuta cruzi*-seropositive individuals. Cardiac damage in infected patients can occur while patients remain asymptomatic for Chagas disease (Ref. 47). Both the onset and progression of Chagas’ cardiomyopathy are found to be associated with the male sex (Refs 51, 52). Males demonstrated greater electrocardiogram (ECG) abnormalities and abnormal echocardiograms when infected, they showed significantly lower left ventricular ejection fraction, greater fibrosis and more prevalent transmural involvement when compared to females with Chagas’ cardiomyopathy (Refs 52, 53). Elevated seroprevalence of *T.c.* was noted in children and women of reproductive age in Veracruz (Ref. 46), but age was not associated with changes in the clinical course or severity of Chagas disease (Ref. 54). Contradictory results, particularly those in non-endemic countries, indicate the need for improved surveillance and profiling of Chagas disease (Ref. 55).

### Human African Trypanosomiasis

HAT shows variable differences in incidence between sexes with some reports suggesting no difference (Ref. 56), other surveys showing male predominance (Refs 57, 58) while others found HAT incidence to be higher among women (Refs 59, 60). The overall incidence of HAT is much lower than CD and leishmaniasis and is recognized to likely have underreported case numbers resulting in inaccurate measurement of disease distribution. Undetected *T.b.* infections may be due to a suboptimal system of case detection utilizing sentinel sites. This lack of detection results in “silent transmission” of disease (Ref. 61). In addition to challenges to surveillance and reporting, the variability of evidence of sex bias in incidence could be attributed to differing sex-specific behaviours in different study areas (Ref. 58).

Currently, there is limited published data available regarding animal models demonstrating sex differences in infection or in disease progression of HAT. Yet, one study in mice found that male mice were more susceptible to infection compared to females who demonstrated increased resistance and lower parasite burden (Ref. 62). Otherwise, mouse HAT models looking at severity and progression did not note sex to be implicated in disease processes. These studies did identify inflammatory cytokines and the role they may play in exacerbating CNS damage during infection, which, although not explored in these studies, are known to be influenced by sex hormones and sex-specific genetics (Refs 10, 11, 29, 63, 64, 65, 66, 67, 68).

In clinical studies of human disease, a 2002 study investigating differences between the genders in cases of human HAT cases conducted in Nioki, Democratic Republic of Congo (DRC) noted adult men were more likely than women to have an abnormal cerebrospinal fluid (CSF) white cell count, indicating increased progression to stage 2 infection involving encephalopathy in males compared to females (Ref. 60). This difference was not observed between infected males and females 0–15 years of age (Ref. 69). One study found that in Nioki, DRC, males had elevated numbers of trypanosomes and white blood cells in the CSF compared to female patients (Ref. 60). However, the study also concluded that male sex is not a risk factor for increased mortality or encephalopathy rates (Ref. 60). Another study found that males were more likely to experience relapses of HAT after treatment, related to males having a higher parasite burden in their CSF (Ref. 70).

These various sex-based trends in human diseases arising from *Leishmania*, *T.c.* and *T.b.* infections are summarized (Table 2). While research is ongoing, in some regions, issues arise from diagnostic limitations and low case numbers present challenges for accurate quantification.

**Table 2.** Sex differences summary – incidence and severity. Summary of the major clinical and epidemiological sex differences for Leishmaniasis Chagas Disease and Human African Trypanosomiasis. Some conflicting reports exist for HAT, so no well-established differences between sexes are reported.

Disease	Incidence	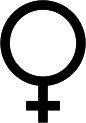	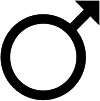
Leishmaniasis	1,000,000/year	Lower incidence Less severe Lower mortality	Higher incidence More severe Higher mortality
Chagas disease	100,000/year	Lower rates of cardiac disease and related mortality	Higher rates of cardiac disease and related mortality
Human African Trypanosomiasis	<1,000/year	No agreed differences inmorbidity/mortality.	No agreed differences in morbidity/mortality.

## Effects of sex hormones on immune response

Sex-related hormones are recently appreciated for their involvement on immune responses which may drive sexual dimorphism in the severity of human *Kinetoplastida* diseases (Figure 1) (Refs 11, 66, 71, 72). The sex hormones of greatest interest are testosterone, progesterone, prolactin and oestrogen. Oestrogen, prolactin and progesterone are most concentrated in females, whereas testosterone is most concentrated in males, and each are especially linked to certain reproductive events (Ref. 73, 74, 75, 76). While each hormone binds to unique receptors, structurally, progesterone, testosterone and oestrogen are related and can be inter-converted via enzymatic reactions and intermediate products (Ref. 77). These hormones have numerous effects throughout the body of all humans (Figure 1), however, it has been generally found that testosterone and progesterone are anti-inflammatory signals, whereas prolactin and oestrogen have pro-inflammatory effects (summarized in Table 3) (Refs 71, 74, 75, 78, 79, 80). These immunomodulatory effects can be seen in relevant cell types to *Kinetoplastida* infections such as innate lymphoid cells (ILCs) and dendritic cells of the skin, among others (Refs 68, 81, 82, 83). Therefore, with their varying concentrations and immune impacts, there is the potential for sex hormones to affect the sex-biased incidence and disease progression of human *Kinetoplastida* diseases.

**Table 3.** Summary of immune response and sex hormones in Humans.

Hormone	Primary Production site(s)	Cognate Receptor	Primary Functions	Immunological Effects	Sources
Testosterone	Testes	Androgen Receptor (AR)	Puberty and promotes testes immuneprivilege	Anti-inflammatory. Promotes IL-10, Th1 and CD8 activation; inhibits NK, TNF-α, IL-6 and IL-1β functions.	(Refs 71, 78)
Progesterone	Adrenal cortex, ovaries, testes, and placenta	Progesterone Receptor (PR)	Maintain pregnancy	Anti-inflammatory. Inhibits NK, macrophage, T lymphocyte cells. Upregulates IL-3, IL-4 and IL-10. Downregulates IFN-γ, TNF-α and IL-2.	(Refs 75, 79, 80)
Prolactin	Pituitary gland, and uterus, thymus, spleen and elsewhere	Prolactin Receptor (PRLR)	Lactation, sleep (circadian rhythm) and homeostasis	Pro-inflammatory. Promotes graft rejection and T cell proliferation.	(Refs 74)
Estrogen	Ovaries	Estrogen Receptor (ER)	Puberty, menstruation and ovulation	Pro-inflammatory. Promotes Th2 signature at low (male physiologic/ menopausal/premenarchial) and high (pregnancy) concentrations. Promotes Th1 signature at moderate concentrations (female physiologic). Elevates pathogen specific humoral response.	(Refs 66, 68, 72, 171, 172, 174, 175, 178, 179, 180, 181, 182, 183, 263)

**Abbreviations:** NK: natural killer cell; IFN: interferon, IL: interleukin, TNF: tumor necrosis factor.

### Testosterone and immunity during human kinetoplastida-caused diseases

Immune modulation plays a critical role in the severity of many diseases; particularly, during kinetoplastid*-*caused human diseases (summarized in Figures 2, 3 and 4). For a general example, testosterone’s immunosuppressive effects on innate immune cells are evident in DCs and ILCs - cells commonly present in the skin and serve as the first line of defence for diseases spread by insect bites. Clinical and experimental studies demonstrate that testosterone suppresses DC activation and pro-inflammatory cytokine production of IL-1β, IL-6, tumour necrosis factor (TNF)-α, while reducing major histocompatibility complex (MHC) class II and co-stimulatory molecule expression (Refs. 84, 85). This is evidenced by enhanced DC responses in hypogonadal men and castrated mice, suggesting testosterone restrains DC-mediated immunity (Ref. 85). In ILC populations, *in vivo* studies of humans and mice show testosterone exhibits significant suppressive effects on ILC2s, which are found in higher numbers in women with asthma compared to men (Ref. 86). Testosterone regulates ILC2 function through direct decrease of ILC2 counts and suppression of ILC2 IL-5/IL-13 production, while also reducing tissue production of ILC2-stimulating cytokines (IL-33 and TSLP). Additionally, testosterone negatively regulates IL-2 receptor-mediated ILC proliferation (Ref. 86). These effects on innate immune cell populations could influence early immune responses during *Kinetoplastida* infections, where rapid cytokine production and immune cell recruitment are crucial for parasite control. The immunosuppressive effects of testosterone on these innate immune populations could help explain the generally worse disease outcomes observed in males during *Kinetoplastida* infections, particularly through delayed or diminished early immune responses that allow for enhanced parasite establishment.

While the immune modulatory power of sex hormones is becoming evident, there are some distinctions with respect to their effect on disease outcomes within kinetoplastid diseases. For example, in *T.b.* infections of all subspecies, it has been observed in rats (Ref. 67), dogs (Ref. 87), boars (Ref. 88), rams (Refs 89, 90) and humans (Refs 91, 92) that serum testosterone declines over the course of the disease and recovery of serum testosterone can be slow even after treatment (Refs. 90, 91). This observation of declining serum testosterone has been made in both men and women (Ref. 89). Although in males, parasitic invasion of the testes has been seen which may contribute to Leydig cell dysfunction and therefore lower testosterone production, the exact mechanism of this disruption is still unknown (Refs 67, 89, 92). Notably, inoculations of male rats with trypanolytic *T.b.* pellet component fractions recapitulated the same loss of testosterone when the fractions included a unique trypanosomal endoprotease as well as undetermined secreted trypanosomal proteins and glycoproteins produced by the parasite (Refs 93, 94). Regardless of the exact cause of the testosterone loss, in the case of *T.b.* infection, testosterone may be protective for patients with HAT as it promotes humoral immune responses via IL-10 and IL-4 stimulation (Figure 2). Antibody responses can kill *T.b.* by complement and other mechanisms *in vitro*, thus accelerating the humoral response development may limit *T.b.*’s ability to establish infection (Figure 2) (Refs 95, 96). Hence, testosterone loss could have a pronounced impact in males on the course of *T.b.* infection.

Meanwhile, testosterone has contradictory actions in *T.c.* infection. On one hand, in rats and mice testosterone declines during *T.c.* infection (Refs 102, 103, 104, 105, 106), but a mechanistic cause has not yet been established. In contrast, there is evidence that *T.c.* infection may be supported by testosterone presence. For example, *T.c.* has endogenous enzymes that can produce testosterone when provided with precursors (Ref. 107). Meanwhile, in mice with *T.c.* infection, testosterone was also found to be advantageous to the parasite as it is necessary for disrupting thymocyte differentiation (Ref. 108). Specifically, thymus epithelial cells expressing androgen receptors (AR) mediate TNF-α and Caspase3-dependent apoptosis of immature thymocytes, a decline of IL-12 levels, and an increase of TNF-α (Ref. 109). Furthermore, in large vesper mice (*Calomys callosus*), testosterone has been shown to be required to achieve high parasitaemia in male mice as testicular surgical removal provides resistance to *T.c.* infection, which was negated when testosterone was supplemented (Refs 110, 111). Mechanistically, this may relate to observations that the castrated (low testosterone) vesper mice had greater percentages of lytic antibodies, more splenocyte proliferation and more nitric oxide (NO) production. All of these events would be protective immune processes (Figure 3) (Ref. 111). Consequently, gender through variation in testosterone levels may be another potential source to explain sexual dimorphism in *T.c.* disease pathologies. Further research is warranted to determine the impact of testosterone addition or loss during mid-late infection in animal models to determine whether this would be a fruitful pathway for investigation to reduce disease burden in people also.

Lastly, testosterone has been studied and reviewed for its role in leishmaniasis (Refs 115, 116, 117, 118). Clinically, during CL and VL with unreported *Leishmania* species, patients showed declines in testosterone levels (Refs 119, 120). However, patients who developed post-kala-azar dermal leishmaniasis (PKDL) had testosterone increase correlated with IgG up-regulation which combined may impact the disease severity (Refs 121, 122). In these PKDL patients, treatment with miltefosine caused a decline in testosterone which may have aided in restoring normal immune function to clear the disease, but the mechanism of this change is not yet clear (Ref. 121). Also, *in vivo* and *in vitro* studies have shown that males and females with dihydrotestosterone (DHT) or testosterone treatment all had worse parasitic outcomes during *L. mexicana* (Ref. 116), *L. major* (Ref. 123), and *Labrus donovani* infections (Refs 124, 125, 126, 127, 128, 129). Moreover, *L. mexicana* promastigote pre-treatment with DHT also stimulated parasite growth, boosted macrophage infection rate and made *Leishmania* more resistant to killing *in vitro* which combined to exacerbate the disease severity of BALB/c mice *in vivo* (Ref. 130). Furthermore, *L. panamensis* infection of hamsters treated with testosterone resulted in increased lesion size and increased levels of detrimental anti-inflammatory cytokines (Figure 4), indicating a distinct immune response to *L. panamensis* when testosterone was added (Ref. 131). Thus, regardless of the exact *Leishmania* species, this data reveals that testosterone is a powerful immune modulator that drives disease outcomes and needs further consideration.

### Prolactin and immunity during human kinetoplastida-caused diseases

Prolactin functions as both a hormone and a cytokine in the immune system, impacting dendritic cells (DCs) and other innate immune cells. Prolactin and its receptors are expressed by multiple immune cell types, including DCs, macrophages and lymphocytes, promoting both endocrine and autocrine signaling (Refs 138, 139). In DCs, prolactin promotes maturation and differentiation by stimulating IL-6 and IL-23 secretion, potentially enhancing their antigen-presenting capacity and stimulating Th1 inflammatory response (Ref. 140). The enhancement of innate immune function may be relevant during early responses to *Kinetoplastida* parasites, where effective DC and macrophage activation is crucial for pathogen control. Additionally, prolactin’s ability to inhibit immune cell apoptosis and promote pro-inflammatory cytokine production could support sustained immune responses during chronic infection phases, though this requires further investigation in the context of *Kinetoplastida* diseases (Ref. 139).

In the context of HAT, Prolactin has not been thoroughly investigated. The few existing studies show that while prolactin release in healthy adults coincides with sleeping hours and with cortisol release, in patients with advanced HAT, circadian rhythm is severely disrupted as is evident through their sleeping patterns and sporadic spikes in cortisol and prolactin release (Refs 141, 142, 143). Among these patients with severe sleeping sickness symptoms, interferon (IFN)-γ was 7–12 times higher than in healthy patients (Ref. 143), possibly suggesting a role in circadian rhythm in regulating inflammatory signalling and suggesting that such IFN-γ release is insufficient to control or counterproductive for *T.b.* late-stage infection, unlike the other *Kinetoplastida* parasites discussed in this review. However, the relationship between circadian prolactin release and immune functions needs further investigation to link and explain these observations more clearly in human patients. Furthermore, the impact of sex differences on prolactin production and impact on disease state has not been explored for HAT.

Meanwhile for American Trypanosomiasis, some have hypothesized that due to prolactin’s role in cardiomyopathies and its role as an autoimmune pro-inflammatory factor, it may contribute to CD (Ref. 144). Since then, it has been observed that during *T.c.* infection, glucocorticoid endocrine signalling increases, meanwhile prolactin production declines (Refs 145, 146, 147, 148). Many immune functions are impacted by loss of prolactin. This is evident as prolactin administration to *T.c.-*infected mice results in elevated lymphocyte proliferation and activation in multiple cell types (CD4+, CD8+, natural killer (NK) and B cells), macrophage activation and NO production, meanwhile, parasite burden declined – all of which are favourable to fight the infection (Figure 3) (Refs 149, 150). More specifically, *T.c.* under normal conditions induces apoptosis of immature T cells in the thymus, but prolactin is both necessary and sufficient to stop this parasite effect (Ref. 151). Thus, prolactin is a vital immune mediator and a potential area for immunotherapy development for Chagas disease.

Finally, in leishmaniasis prolactin has been understudied and yielded conflicting findings. It has been hypothesized that prolactin may be crucial in *Leishmania* immunity due to its correlation to levels of inflammatory chemokines such as macrophage inflammatory protein (MIP-1a) (Ref. 136) and monocyte chemotactic protein (MCP-1) (Ref. 152). Meanwhile, a study on the levels of serum hormones during localized cutaneous leishmaniasis in Brazil (likely due to either *L. braziliensis* or *L. amazonensis*) documented a reduction in serum prolactin levels for both male and female localized CL patients (Ref. 153). Notably, this study found excessive prolactin levels correlated inversely to IFN-γ production and correlated positively to increased lesion diameter (Ref. 153). Finally, prolactin likely is pivotal in *L. major* immunity as the prolactin-inducible protein (PIP) is necessary for optimal CD4+ T-cell proliferation, Th1 differentiation, IFN-γ production, NO production in macrophages and ultimately *L. major* killing (Figure 4) (Ref. 154). Moreover, PIP-deficient macrophages were more susceptible to *Leishmania* even when IFN-γ and lipopolysaccharide (LPS) stimulated (Ref. 154). Thus, the role of prolactin in leishmaniasis immunity especially over various disease presentations and parasite species requires further investigation.

### Progesterone and immunity during human kinetoplastida-caused diseases

Progesterone has been demonstrated to play a role in regulation of innate immune cells. It has a known impact on DCs, as they contain progesterone receptors, however exact mechanisms are still under investigation (Ref. 155). Evidence suggests progesterone modulates DC function by promoting DC differentiation and increasing costimulatory factors such as MHC-II, CD40, CD54 and CD86. Also, progesterone can induce a shift towards an anti-inflammatory cytokine profile with enhanced IL-10 and reduced TNF-α production, although this may be less prominent in mature DCs (Ref. 156). For innate lymphoid cells, progesterone’s effects, studied primarily in pregnant women, show tissue-specific variation. In reproductive tissues, high progesterone promotes ILC2-mediated responses (Ref. 157) while in the lung, progesterone enhances ILC2 responsiveness to IL-33, leading to enhanced IL-13 and chemokine release (Ref. 158). However, the systemic effects of progesterone on ILCs in the context of parasitic infections, warrants further investigation.

Progesterone has not been explored in the context of *T.b.* human infection. But, during *T.c.*-infection of humans and mice with cardiomyopathy, progesterone has been seen to alter gene expression of hub genes which has cascading effects on innate immunity and elsewhere throughout the body (Ref. 159). This mechanism may relate to the observed reduction of parasite burden during progesterone supplementation for ovariectomized *Calomys callosus* infected with *T.c.* (Ref. 160). Also, *T.c.*-infected rats treated with melatonin saw reduced progesterone levels which may have contributed to reduction of IL-1β and TGF-β levels (Ref. 161). Finally, mice infected with *T.c.* of distinct genotypes revealed that distinct immune responses are generated regardless of equivalent serum progesterone levels, indicating the strong role parasite genotype also plays in potential patient outcomes (Ref. 162).

Also, limited investigations into progesterone during leishmaniasis have been conducted. Mechanistically, one study demonstrated that progesterone acts through the glucocorticoid and progesterone receptors on macrophages to reduce IL-12 and NO production, thus limiting killing of *Labrus donovani in vitro* (Figure 4) (Ref. 163). Moreover, clinical observations of un-spayed, nonpregnant dogs with natural VL infection showed that dogs with high serum progesterone were more likely to have chronically high parasite and inflammatory infiltration into the mammary glands (Ref. 164). Further evidence specific to progesterone involvement in leishmaniasis is lacking, however some prior studies regarding the impact of pregnancy (which requires high progesterone) on *Leishmania* infections have been reviewed (Refs 115, 116). Briefly, pregnant mice were found to have elevated burdens of *L. major* and elevated Th2 immune responses compared to non-pregnant mice (Refs 165, 166, 167), however during hamster *L. panamensis* CL infection, pregnant mice had lower parasitaemia and improved macrophage NO production (Ref. 168). Thus, progesterone requires more detailed investigation to fully understand its role in leishmaniasis for pregnant and nonpregnant patients with various *Leishmania spp.*

### Estrogen and immunity during human kinetoplastida-caused diseases

Estrogen is primarily produced by the ovaries and has a wide range of impacts throughout the body as nuclear oestrogen receptors (ERs) are expressed by a range of cell types (Refs 169, 170). There are four identified human oestrogens that act on oestrogen receptors with varying affinities. Oestradiol (E2) has the highest affinity for all ER types and is produced most in women of reproductive age (Ref. 166). Estrone (E1) is created primarily in adipose tissue and adrenal glands and is the prominent oestrogen subtype in post-menopausal women (Refs 68, 170, 171). Estriol (E3) is usually only seen during pregnancy as it is secreted by the placenta (Refs 68, 172). Finally, oestetrol (E4) is a foetal oestrogen only detectable during pregnancy (Refs 68, 170, 171). The impact of oestrogen on each cell type is dependent on the intracellular response cascade and has different effects depending on the receptor and cell type. In the immune system, oestrogen exhibits a variety of impacts on both innate and adaptive immunity. Oestrogen acts on B cells, T cells (cytotoxic and helper), dendritic cells, natural killer cells, monocytes and haematopoietic stem cells through three subtypes of ERs: ERa, ERb and G protein-coupled ER1 (GPER1) (Refs 68, 171, 172, 173, 174). The overall effect of these ERs impacts immune cell activation and function such as the promotion of an increase in total serum antibodies (Refs 68, 175). Overall, oestrogens can have a wide range of effects depending on the reproductive status of the woman and oestrogen concentration, the microenvironment, the cell and tissue types involved and the ER expression levels in the involved cells (Refs 175, 176, 177, 178).

Oestrogen’s effects on innate immunity show complex patterns that vary with oestrogen concentration and cellular environments. In dendritic cells, oestrogen acts on ERα to regulate DC development depending on the cytokine environment. Oestradiol acts on DC progenitor cells at physiologic levels in females, it promotes granulocyte-macrophage colony-stimulating factor (GM-CSF) mediated differentiation of DCs while inhibiting Flt3L-mediated DC differentiation (Ref. 179). Furthermore, ERα signalling modulates DC function by influencing cytokine production. DCs from ERα-deficient mice show reduced production of IL-6, IL-23, IL-12 and IL-1β in response to TLR stimulation (Ref. 179). However, at high serum oestrogen concentrations such as those during pregnancy, oestrogen can act as an anti-inflammatory modulator by promoting a tolerogenic DC phenotype with increased expression of inhibitory molecules and regulatory cytokines (Ref. 68). Evidence as to the relationship between oestrogen and ILC2s appears more limited, with most tissue-resident ILC2s lacking significant expression of oestrogen receptors. While uterine ILC2s express oestrogen receptors and respond to oestradiol through a mechanism of altered gene expression, this may be related to oestrogen’s effects on the uterine environment rather than direct immune cell regulation. In the lung, where ILC2s play important roles in inflammation, there is little evidence for direct oestrogen regulation of ILC2 numbers or function under normal conditions (Ref. 83). These complex regulatory patterns of oestrogen on innate immune cells may help explain the generally enhanced immune responses observed in females during *Kinetoplastida* infections, while also contributing to increased susceptibility to autoimmune conditions.

Additional immune mediation impacts of oestrogens include shifting Th2:Th1 ratio, elevating antibody levels and modulating expression of cytokines and inflammatory mediators (Refs 175, 178). Of note, these pro-inflammatory actions can be harmful as high affinity autoreactive B cells were found in increasing levels in response to oestrogen, which is a potential mechanism for autoimmune disease in females (Ref. 180). Moderately high levels of oestrogen, such as those in reproductive age females, enhance the Th1 response (Figure 1) (Refs 66, 181). Studies demonstrate that at normal female physiological levels, oestrogen enhances IFN-γ expression by Th1 cells in both humans and mice (Refs 181, 182). In the presence of very low, such as those seen in males, and very high oestrogen levels, such as those seen during pregnancy, Th2 differentiation and cytokine expression is favoured (Refs 68, 175, 178, 181, 183). One study showed that at very high concentrations, oestrogen induces a shift towards a Th2 signature, inhibiting Th1 cytokines, including IL-12, TNF-α and IFN-γ, and stimulate production Th2 cytokines, including IL-10, IL-4 and TGF-β (Ref. 178). This, however, is only supported during especially high levels of oestrogen, seen physiologically during pregnancy (Ref. 178). Other studies support the finding that overabundance of oestrogenooestrogens shifts the T-cell profile to favour Th2 differentiation and cytokine production (Refs 72, 184, 185). Thus, oestrogen levels in pregnant women exacerbate Th1-mediated diseases and inhibit Th2-mediated autoimmune diseases, but oestrogen levels in reproductive aged non-pregnant females compared to males have a protective effect against Th1 mediated diseases such as viral, bacterial and eukaryotic infections. Given this variability in immune effects, oestrogens also may contribute to the sexual dimorphism seen in HAT, Chagas disease and leishmaniasis; Th1 cytokines – IL-12, IFN-γ TNF-α – are known to be implicated in the immune response and elimination of these pathogens (Figures 2, 3, and 4), while Th2 cytokines – IL-10, IL-4 and TGF-β – are implicated in *Leishmania* survival (Figure 4).

Oestrogen and its impacts on leishmaniasis have been well studied to date. Oestrogen has been shown to have a protective effect against *Leishmania* infection and the severity of the disease (Refs 123, 186, 187, 188, 189). This is supported by studies where sex-differences are significantly less prominent or absent before puberty (Refs 186, 187). Moreover, adult females exhibit smaller *Leishmania* skin lesions compared to pre-pubescent juveniles (Refs 186, 187). Overall, adult female infections showed to be less severe and less frequent than those in juvenile females or males related to the reduced Th2 response in adult females (Ref. 10). Additionally, in mice, exogenous E2 administration was shown to induce leishmanicidal activity in macrophages through NO modulation (Ref. 190). As seen in Figure 4, *Leishmania* species favour elimination in the presence of Th1 cytokines, M1 cytokines and pro-inflammatory mediators such as NO. Thus, given oestrogen’s ability to enhance these at physiologic levels in non-pregnant females, oestrogen likely works through a variety of these mechanisms to enhance the immune response in females and favour parasite elimination thus contributing to sex-bias seen in leishmaniasis.

Despite systemic mammalian oestrogen having an overall protective effect against leishmaniasis infection, selective oestrogen receptor modulator (SERM) therapy with Tamoxifen, which competitively inhibits oestrogen action, is effective in inducing parasiticidal activity in infection by certain *Leishmania* spp. (*L. amazonensis, L. braziliensis, L. major, L. chagasi* and *L. donovani*) *in vitro*, *in vivo* and clinical trials (Refs 187, 188, 189). Tamoxifen’s therapeutic benefit in leishmaniasis stems from its highly selective interference of oestrogen-mediated sphingolipid production in *Leishmania* resulting in parasite death due to cell membrane destruction (Refs 191, 192). Given the selective nature of SERMs, only impacting a very specific subset of ERs, this effect on sphingolipid production in the parasite occurs without impacting all oestrogen receptors of the host, thus limiting off target negative side effects (Refs 191, 192, 193). This highlights the complex nature of oestrogens and oestrogen receptors making it difficult to rule the hormone’s effects as either protective from or supportive of infection. Certain mechanisms such as its impact on sphingolipid production is supportive of infection and thus used as a drug target (Refs 187, 188, 189), while other cytokine and immune modulatory mechanisms of oestrogen such as Th1 promotion, are protective against most forms of leishmaniasis (Refs 116, 123, 187, 188, 190).

The relationship of *T.c.* and *T.b.* infections to oestrogen is less well documented possibly related to the lack of evidence of sex bias in these diseases resulting in a relative lack of investigation into this topic. As demonstrated in Figure 3, *T.c.* elimination is supported by reactive nitrogen species, cytokines from macrophages, parasite-specific antigens and Th1 cytokines such as IL-12 (Ref. 194). Thus, oestrogen’s role in shifting the Th cell profile is likely not influential in contributing to any sex differences in Chagas disease. More so, oestrogen likely impacts immune response to *T.c.* infection through Th1 cytokines IL-12/IFN-γ and antibody production and reactive nitrogen species (RNS) (Refs 194, 195). Moreover oestrogens play a role in promoting RNS (Ref. 196) and the elimination of *T.c.* in acute phase disease (Ref. 197), along with the role of reactive nitrogen species in *T.c.* elimination (Ref. 51). However, there is not any available evidence confirming the direct effects of oestrogens on acute phase RNS during *T.c.* infection.

There is no available literature on oestrogen’s impact on the immune response to *T.b.*, nevertheless, based on what is known of the immune response to *T.b.* and oestrogen’s immunomodulatory effects, we could hypothesize that oestrogen would favour of the Th1 and M1 response over the Th2 and M2 response. While the M1 and Th1 responses are important in limiting parasitaemia in acute infection, it is key to host survival that the immune response favours an M2 response during an advanced infection to avoid excess inflammatory cytokines that cause host tissue damage and cell death (Figure 2) (Ref. 198). Studies show a relationship between inflammatory cytokine levels during stage two HAT, indicating CNS infection, and subsequent risk of nervous system damage (Refs 10, 11, 29, 63, 64, 65, 66, 67, 68). Given this, there is the potential that oestrogen related pro-inflammatory cytokines could exacerbate tissue damage in the CNS during this stage of infection. Although females tended to have lower parasite concentrations (Refs 62, 69), pro-inflammatory states may cause adverse disease outcomes in women. Studies have also found that the inability to upregulate alternative macrophage activation and limit classical activation created increased susceptibility to infection and death of infected mice earlier on in the disease (Ref. 198). Still, further research on how oestrogens impact macrophages, and other innate and adaptive immune cells and regulate gene expression during the immune response to *T.b.* is yet to be described.

In summary, the sex hormones testosterone, progesterone, prolactin and oestrogen modulate immune responses against *Leishmania*, *Trigonoscuta cruzi* and *T. brucei* parasites through varied and complex mechanisms. While more research is required in many areas, current evidence suggests testosterone tends to exacerbate disease caused by kinetoplastids, although it may be protective in the case of HAT, prolactin is important for controlling Chagas disease, progesterone’s role is unclear, and oestrogen often enhances immune clearance of *Leishmania* but has less characterized impacts for Chagas disease and HAT.

## Genetic and gene expression differences in immune response

Numerous clinical studies show that there are substantial sex-based disparities in the prognosis of several infectious and immune diseases (Refs 199, 200, 201, 202). Females typically have stronger humoral and cellular responses to infection or antigenic stimulation than males (Refs 64, 203). This characteristic can provide protection against various infections, although in some cases an exacerbated immune response has been observed as a potential risk factor during immunopathological changes or treatment, such as immune restoration diseases in HIV-TB co-infected patients (Refs 204, 205). While there are general sex-differences in innate immune response, female and male genetic influences on immune responses during parasite infection are pathogen-specific (Ref. 206). *Trichomonas vaginalis* and *Toxoplasma gondii* resistance was shown to be higher in males, while leishmaniasis resistance was found to be higher in females (Refs 207, 208). Males are more likely to be affected by VL (Ref. 25, 36) and certain CL infections than females (Refs 30, 31, 32, 33, 42). While in individual infectious diseases, some differences according to gender have been documented, determining what originates from genetic differences between genders can be thorny. For example, genetic differences between genders could manifest as different rates of beneficial or harmful alleles, different gene expression patterns or different allele numbers, particularly for genes related to the sex chromosomes; yet genetic differences also combine to produce different hormone levels discussed previously which have their own cascading effects. We will distinguish these sources of genetic differences here. For example, the X and Y chromosomes’ genetic material controls the expression of traits with non-hormonal bias toward either sex (Refs 63, 65, 209). Many immune-related miRNAs are also found on the X chromosome, among immune-related genes such as toll-like receptor (TLR)-7 and interleukin-1 receptor-associated kinase-1 (IRAK-1) (Ref. 63). Some portion of the X chromosome remains active, which may boost sex-specific gene transcription, despite the fact that X-chromosome inactivation (or silence of one X chromosome) in women is supposed to result in dosage compensation of X-linked genes (Refs 210, 211).

**Figure 5.**
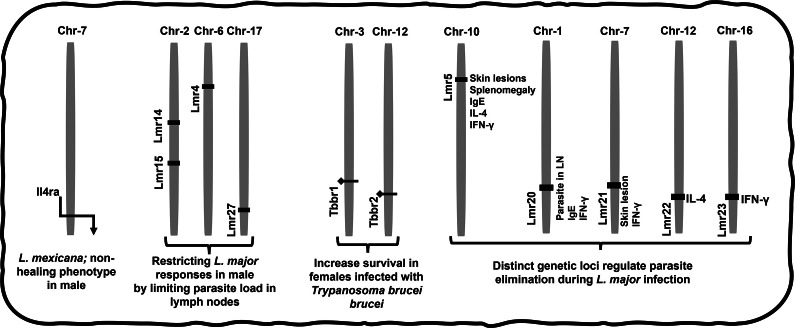
Parasite regulation by differential genetic loci and chromosome. The chromosomal gene loci indicated are directly associated with kinetoplastid disease outcomes and differences in their roles have been observed by sex. Additional genetic products such as proteins, hormones and enzymes also exist which are interdependent in their influence on immune responses, and which may also show differences by sex. **Abbreviations:** Chr: Chromosome, Lmr: *Leishmania major* response, Tbbr: *Trypanosoma brucei brucei* response.

In contrast, Y chromosomes affect infection susceptibility, immunological gene expression and regulation (Ref. 209). Further studies have also observed more specific gene-pathogen associations that are differentially apparent by gender. This includes the genes Lmr4 (chromosome; Chr-6), Lmr14 (Chr-2), Lmr15 (Chr-2) and Lmr27 (Chr-17) that control anti-*L. major* responses in male BALB/c mice by limiting parasite load in lymph nodes (Figure 5) (Refs 212, 213). Similarly, CD4+ T cells’ specific Il4ra gene on Chr-7 promotes the *L. mexicana*-mediated non-healing phenotype in male BALB/c mice (Ref. 214), and genes Tbbr1 (Chr-3) and Tbbr2 (Chr-12) help in increasing survival in BALB/c females infected with *T. b. brucei* (Ref. 215). Tbbr1 is only evident in females, and Tbbr2 expresses higher in females than males. Loci on Chr-17 and Chr-5 determine susceptibility to *T.c.*, however, the role of Chr-5 in sex differentiation remains unelucidated. Because of its overlap with Lmr22 and interaction with Lmr5, which regulates IL-4 release in the serum of *L. major*-infected mice, Tbbr2 may also play an important role in regulating *L. major* (Ref. 216). On a similar note, DBA/2 mice infected with *L. mexicana* showed two different clinical manifestations: ulcerative lesions in males that did not heal and healing lesions in females (Refs 217, 218). Scl2 is the gene has been identified to regulate this (Ref. 218). Due to antigen-specific vulnerability, these various genes across several chromosomes may affect immune responses differently between genders and need more investigation to elucidate their regulation further.

## Socio-cultural Influences

Disease-related stigma denies impacted individuals of social acceptance, reduces economic opportunities, allows for discrimination and greatly influences health-seeking behaviour by creating a barrier to care (Ref. 219). Certain diagnoses can lead to stigmatizing behaviours and discrimination including housing refusal, verbal abuse, gossip and self-stigma, creating social devaluation and negative emotions for an individual (Ref. 219). While stigma affects men and women, women have reported avoidance of healthcare services due to fear of stigmatization by their communities or their providers for a multitude of diseases, contributing to worsened health outcomes and underreporting of disease (Ref. 219).

Active and scarred CL lesions create a greater stigma for women than men (Ref. 220). In rural Morocco, Yemen and Afghanistan, CL facial scars cause stigmatization that hinders marriage prospects and harms the self-esteem of young women (Refs 220, 221). Additionally, scarring can decrease education and employment opportunities (Ref. 220). Because women typically perform unpaid labour in these areas, they face a greater financial barrier to care than men (Ref. 221). Furthermore, CL-afflicted women in Colombia and Brazil reported avoiding healthcare clinics due to distrust, while pregnant or nursing women in Ecuador feared seeking treatment due to drug toxicity and potential damage to their child, and Afghani women reported restrictions to care due to the required presence of a male in clinic with the patient, and the requirement that women be treated by a female provider (Ref. 220). Additionally, for every Colombian man who sought treatment, 13 women sought home remedies, leading to decreased reporting and worsened outcomes for women (Ref. 220).

CD infection, despite its lack of association with any physical attributes, is also associated with poverty, stigma and decreased quality of life (Ref. 222, 223). Chagas may be used as a reason to fire an individual from work and prevent them from passing pre-hiring physical examinations in Brazil (Ref. 222). Employment also acts as a deterrent to seeking treatment for CD in women. In Italy, Bolivian female migrants often work in caregiving roles, regardless of their education, and seeking care for CD required them to take time off from work and was associated with stigmatization by employers that believed CD to be contagious, decreasing access to and seeking of healthcare for women, contributing to underreporting (Ref. 224). In contrast to some other findings, in Barcarena, Pará and Brazil, gender was not found to be a statistically significant factor in *T. c.* infection, indicating that in those communities infection negatively impacts employment opportunities and healthcare-seeking for both men and women (Ref. 225).

HAT does not demonstrate differences in stigmatization between men and women, likely due to HAT being endemic in areas where it is studied, allowing communities to become familiar with the disease (Ref. 222). However, HAT is associated with stigmatization in both women and men due to potential resultant neurological impairments and performance impediments that could lead to individuals facing gossip, mockery and loss of social status, deterring individuals from seeking care (Ref. 222, 226). Moreover, in the DRC (Ref. 226), neurological symptoms associated with HAT are believed to be associated with witchcraft by women or dark forces (Ref. 226). While women and men with HAT are both stigmatized, only women are believed by some communities to be responsible for the disease (Ref. 226).

While stigma may impact the rate that individuals seek treatment, other behaviours may also impact the rate of disease incidence among genders. Risk-taking behaviours such as risk-taking behaviours could also impact the rates of disease among each gender. Risk-taking behaviour includes unsafe sexual practices, rule breaking, self-injurious behaviours and aggressive violent or behaviours (Ref. 227) and given their associations to various disease states, differences in these behaviours between males and females can contribute to gender differences in disease occurrence and outcomes. Males take part in risk-taking behaviours at a greater rate than females. This trend is attributed to these behaviours providing males with greater reproductive success and enhancement of social status from an evolutionary psychology perspective; males faced stronger reproductive competition than females, creating a riskier sexual selection process for males (Ref. 227). Therefore, males are more likely to perceive the benefits of risky-behaviours, while females are more likely to perceive the associated risks (Ref. 227).

In the context of leishmaniasis, the impact of risk-taking behaviours is beginning to be observed. A study of leishmaniasis in Israel suggests risk-taking behaviours, specifically travel to endemic regions with increased outdoor exposure, led to a high incidence of males contracting *Leishmania spp.* not known to be endemic in Israel (Ref. 228). Risky behaviours, such as working in a specific area, refusal to seek treatment, travel to endemic areas and participation in outdoor activities leading to increased vector exposure contribute to the differences in infectious disease incidence between men and women such as that seen in leishmaniasis (Ref. 220). Furthermore, in Suriname, risky behaviours such as working in the sectors of gold mining, lumber or construction increased one’s risk of CL and were associated with masculinity (Ref. 220). In Colombia, where risk-taking is associated with responsibility, CL was linked to serving in the army, which predominantly affected males (Ref. 220). In Morocco, however, where women were primarily responsible for farming, handling manure and caring for cattle, they were placed at higher risk of CL infection than men (Ref. 220). These differences may contribute to variable incidence reports and risk calculations of sex in different communities.

Despite areas endemic to CD also having gender roles associated with jobs, there was no significant difference in infection between men and women, indicating there is likely equal exposure to infection (Ref. 225, 229). However, in Bolivia, men were found to be less likely to seek help when infected to avoid appearing weak and potentially losing their jobs which could mean an underreporting in males and thus underestimation of sex-bias towards male infection for CD (Ref. 230, 231). Women, however, were more likely to utilize healthcare services related to their reported desire to stay alive and healthy to provide care for their children (Ref. 231). This finding for CD contrasts with that in Colombia for CL. While women in Bolivia were more likely to seek treatment for CD than men, women in Colombia were far more likely to try multiple home remedies first for CL (Ref. 229, 220). Although not confirmed, this could be attributed to stigmatization associated with CL facial scars in women (Ref. 220). CD, on the other hand, does not have an association with physical attributes and does not create this stigma between men and women (Ref. 222).

Research suggests regardless of gender, communities perceived all individuals to be at risk for HAT (Ref. 226). Some studies reported that activities with the most significant gender differences were not significant predictors of HAT risk (Ref. 57). Other studies reported activities such as cleansing rituals in Uganda and Kenya, as well as circumcision in Uganda, were the most important cultural risk factors for HAT infection due to increased exposure to tsetse flies (Ref. 232). Male-associated behaviours such as poaching, honey-gathering and land clearing have also been associated with increased HAT incidence (Ref. 233). Thus, strong gender differences in stigmatization and risk-taking behaviour associated with leishmaniasis likely contribute to the gender bias seen in the disease. Meanwhile, less prevalent gender differences seen with CD and HAT may be related to the lesser differences in incidence rates between males and females for these diseases.

## Expert and topical summary

Leishmaniasis, Chagas disease and Human African Trypanosomiasis are neglected tropical diseases caused by kinetoplastid parasites that exhibit sex differences in incidence and disease severity. Leishmaniasis shows a clear male bias, with higher incidence, morbidity and mortality in males compared to females. This is influenced by sex hormones, genetics and gender-related behaviours. In contrast, CD and HAT show less consistent or minimal sex differences in outcomes. The reasons for variability in sex bias between the diseases are not fully understood but likely involve differing interactions between the immune response and sex-related factors such as genetics, hormones and behaviours. More research is needed to identify and understand the specific factors driving increased susceptibility and disease severity in males or females for each pathogen, particularly to understand HAT and CD which have little information available in this area. While the differences between males and females in Chagas and HAT disease risk and outcomes are not fully clear, continuing research in this area could have implications for disease management, treatment, epidemiology and control. Thus, the patient’s sex remains an important factor to consider in the context of these neglected tropical diseases.
